# Molecular docking analysis of beta-caryophyllene with IRS-1, cSrc and Akt

**DOI:** 10.6026/97320630017916

**Published:** 2021-11-30

**Authors:** Vadivel Mani, Manikandan Balraj, Gayathri Venktsan, Jagatheesh Soundrapandiyan, Revathi Kasthuri, Anandhi Danavel, Shyamaladevi Babu

**Affiliations:** 1 Department of Biochemistry, Arunai Medical College & Hospital, Tiruvanamallai-606603, Tamilnadu, India; 2 Department of Physiology, Konaseema Institute of Medical sciences and research foundation, Amalapuram, East Godavari Dt-533201, Andhra Pradesh, India; 3 Department of Physiology, Krishnadevaraya college of dental sciences and Hospital, Bangalore, Karanadakha, India; 4 Department of Research, Meenakshi Academy of Higher Education, Chennai-600078, Tamilnadu, India; 5 Department of biochemistry, Meenakshi Ammal Dental College & Hospital, Chennai-600095, Tamilnadu, India; 6 Department of Biochemistry, Saveetha Dental College and Hospitals, Saveetha Institute of Medical and Technical Sciences, Saveetha University, Chennai, Tamil Nadu, India

**Keywords:** Diabetes mellitus, insulin resistance, Beta-Caryophyllene, molecular docking analysis, IRS-1, Akt

## Abstract

Diabetes mellitus (DM) is a common metabolic illness defined by hyperglycemia caused by insufficient production or absent of pancreatic insulin, with or without concomitant insulin action impairment. Hence, novel problem-solving approaches for assessing early metabolic diseases, notably insulin resistance, are urgently needed. Screening of natural compounds for drug discovery to combat diabetes is common in modern medical research and development. Therefore, it is of interest to document the molecular docking analysis data of beta-Caryophyllene, a naturally occurring sequiterpene with the downstream insulin signaling molecules such as IRS-1, cSrc and Akt for the management of type-2 diabetes. The molecular docking analysis data of beta-caryophyllene with the insulin downstream signaling molecules such as IRS-1, cSrc and Akt reveals its ability and further studies are needed to elucidate its complete mechanism of action against type-2 diabetes.

## Background:

Diabetes mellitus (DM) is a common metabolic illness defined by hyperglycemia caused by insufficient production or absent of pancreatic insulin, with or without concomitant insulin action impairment [1]. The IRS, PI3K-Akt pathway is activated, which leads to increased glucose absorption by skeletal muscle cells and adipose tissue, as well as changes in a variety of physiological processes [2]. Insulin attaches to insulin receptor (IR) located on the surface of cell membranes, which causes the receptor to autophosphorylate. Insulin binding phosphorylates a tyrosine residue in insulin receptor substrate-1/2 (IRS-1/2), an intracellular post-signaling protein. IRS serve as a docking site for phosphatidylinositol 3-kinase (PI3K), which activates Akt/protein kinase B, allowing intracellular GLUT4 to enter the plasma membrane. The IRS, PI3K-Akt pathway is activated, which leads to increased glucose absorption by skeletal muscle cells and adipose tissue, as well as changes in a variety of physiological processes [3]. Excessive consumption of a high-fat diet has been found to cause obesity, which may lead to insulin resistance in target tissues [4]. Insulin signalling is disrupted at several levels during insulin resistance, resulting in decreased glucose absorption in insulin-sensitive peripheral tissues [5]. Hence, novel problem-solving approaches for assessing early metabolic diseases, notably insulin resistance, are urgently needed [6]. Screening of natural compounds for drug discovery to combat diabetes is common in modern medical research and development [7]. Beta-caryophyllene is a naturally occurring sequiterpene found in cannabis as well as a variety of culinary herbs and spices and has a variety of biological actions, including antioxidant, anti-inflammatory, and anti-lipidemic properties [8]. Therefore, it is of interest to document the molecular docking analysis data of Beta-Caryophyllene with the downstream insulin signaling molecules such as IRS-1, cSrc and Akt for the management of type-2 diabetes.

## Methodology:

### Docking study using Auto Dock suite:

Functionality of receptor (proteins) was determined by their 3D structures which are important for all docking studies. 3D structure 1IRS, 1AO7 and 3QKM (IRS-1, cSrc and Akt) already been predicted in previous course of work in Protein Data Bank. Predicting receptor- ligand interactions is critical to success in many therapeutic and pharmacological research areas such as antibody modeling, many signal transduction pathways, identification of peptide, enzymes, protein inhibitors or activators for drug discovery field. The natural ligands selected through literature search for this study using AutoDock4.0 for virtual screening. Gasteiger partial charges were added to the ligand atoms. Nonpolar hydrogen atoms were merged and rotatable bonds were defined. Docking calculations were carried out on the receptor models. Essential hydrogen atoms, Kollman charges, and solvation parameters were added with the aid of AutoDock tools [9]. Affinity (grid) map of 60 x 60 x 60 angstrom grid points and spacing were generated using the auto grid program. AutoDock parameter that was set distance-dependent dielectric functions was used in the calculation of van der Waals and the electrostatic term, respectively. Docking simulations were performed using the Lamarckian genetic algorithm [10]. MGL tools are used to create at The Molecular Graphics Laboratory of the Scripps Research Institute for representation and examination of molecular structures.

### Active site prediction:

The Three dimensional (3D) targeted three receptor crystal stones (PDB ID: 3QKM) Spirocyclic sulfonamides as AKT inhibitors, (PDB ID:1IRS)Irs-1 Ptb Domain Complexed With A Il-4 Receptor Phosphopeptide, Nmr, Minimized Average Structure, (PDB ID:1AO7) Complex Between Human T-Cell Receptor, Viral Peptide (Tax), and Hla-A 0201were retrieved from the RCSB Protein Data Bankis a freely available online database (http://www.rcsb.org/pdb/home/home.do). Before going to docking study we use Computed Atlas of Surface Topography of proteins (CASTp) provides an online resource for locating, delineating and measuring concave surface regions on 3D structures of proteins. These include pockets located on protein surfaces and voids buried in the interior of proteins. The measurement includes the area and volume of pocket or void by Solvent accessible surface model Richards' surface and Molecular surface model Connolly's surface. CASTp can be used to study surface features and functional regions of proteins. CASTp includes a graphical user interface, flexible interactive visualization, as well as on-the-fly calculation for user uploaded structures. CASTp is updated daily and can be accessed freely on the World Wide Web at http://cast.engr.uic.edu [11]. Finally we predict the flexible Active site for given receptor. To minimize energy, the predicted structures were refined by removing the water molecules and co-crystal ligands of the target proteins. Finally, it was used for molecular docking simulation.

### Ligand retrieval and preparation:

The chemical structure of the phyto-ligands was retrieved from the PubChem database which is available at NCBI (http://www.pubchem.ncbi.nlm.nih.gov).

1) PubChem CID: 5281515

2) Name of the compound: beta-Caryophyllene

3) Molecular Formula: C15H24.

Beta-caryophyllene is a sequiterpene in which the stereocentre adjacent to the exocyclic double bond has S configuration while the remaining stereocentre has R configuration. It is the most commonly occurring form of beta-caryophyllene, occurring in many essential oils, particularly oil of cloves. It has a role as a non-steroidal anti-inflammatory drug, a fragrance, a metabolite and an insect attractant. It is an enantiomer of a (+)-beta-carophyllene. The 2D conformations of the phyto-ligands were downloaded in SDF format and converted into PDB format. The structural optimization was performed using Discovery Studio visualizer. Thus, the obtained chemical structures were used for further docking analysis in this study.

### Molecular docking study:

The molecular docking simulation was performed with the targeted receptors and phyto-ligands using Autodock 4.2 by employing Lamarckian genetic algorithm. Ligand molecules were added with hydrogen atom and gasteiger charges were assigned. Grid box was delineated on binding pocket of target proteins and the grid points were expanded in all directions to include the binding region was determined by tethering ligands to target proteins with highest binding energy (kcal mol−1). AutoDock/Vina was employed for docking using protein and ligand information along with grid box properties in the configuration file. AutoDock/Vina includes local search global optimizer [12]. During the docking procedure, both the protein and ligands are considered as rigid. The results less than 1.0 Å in positional root-mean-square deviation-RMSD was clustered together and represented by the result with the most favorable free energy of binding. The pose with lowest energy of binding or binding affinity was extracted and aligned with protein structure for future analysis.Grid Map File Support. Both the AD4 and Vina programs calculate intermolecular interactions by performing trilinear interpolations of grid maps preprocess on the target structure [13]. Vina also uses the target structure to perform a post processing minimization of the docking, the given receptor as well as the ligands. In AD4, maps are pre calculated using a separate program (AutoGrid2) prior to docking and loaded at runtime, while Vina calculates them on-the-fly prior to running the MC searching process. The availability to accessible grid map files generated by AutoGrid provided the foundations for a number of specialized methods, such as the zinc-coordination potentials in the AutoDock4Zn force field, biasing docking using information from molecular dynamics (MD) simulations in AutoDock-Bias and the integration of Grid Inhomogenous Solvation Theory GIST [14] in AutoDock-GIST. GIST is a method to analyze MD simulations and characterize thermodynamic properties of water molecules. It provides a more accurate representation of water molecule interactions with the receptor within ligand-binding sites, but at the expense of a much higher computational cost compared to implicit solvent models. The pose of 2D and 3D ligand-to-target interactions with high binding energy was extracted and visualized using Discovery Studio.

## Results and Discussion:

The active sites of proteins were predicted by using online tool CASTp. For these 3 proteins about 187 binding pockets were predicted, using them the two top pockets were shown in Figure 1(see PDF). The binding cavity of volume and area are shown in Table 1(see PDF). The amino acid that resides in this cavity may play a major role in binding of ligand molecules during docking analysis. The protein is prepared in the Autodock software and output file were taken as source for predicting the active site of the proteins. The protein's active site region was docked with a beta-caryophyllene compound. The grid option of the autodock tool was then used to generate a grid box around the active site of the receptor. The top hit with the same ligand compound was found in the three proteins that were chosen and docked with the beta-caryophyllene compound. Table 2(see PDF) shows a summary of the docking score and docking energy for the docked compounds. Figure 2,3 and 4(see PDF) depicts the interactions between the beta-caryophyllene and target proteins.

## Conclusion:

The molecular docking analysis data of beta-caryophyllene with the insulin downstream signaling molecules such as IRS-1 cSrc and Akt reveals its ability and further studies are needed to elucidate its complete mechanism of action against type-2 diabetes.
